# Implementation of transoperative and immediate postoperative nursing diagnoses in the computerized management system^*^

**DOI:** 10.1590/1980-220X-REEUSP-2022-0123en

**Published:** 2023-04-14

**Authors:** Aline Fritzen, Danielle Pletes dos Santos, Bianca Silva da Rocha, Marielli Trevisan Jost, Rita Catalina Aquino Caregnato, Graciele Fernanda da Costa Linch

**Affiliations:** 1 Universidade Federal de Ciências da Saúde de Porto Alegre, Porto Alegre, RS, Brazil.

**Keywords:** Nursing Process, Operating Room Nursing, Nursing Diagnosis, Nursing Record, Electronic Health Records, Proceso de Enfermería, Enfermería de Quirófano, Diagnóstico de Enfermería, Registros de Enfermería, Registros Electrónicos de Salud, Processo de Enfermagem, Enfermagem de Centro Cirúrgico, Diagnóstico de Enfermagem, Registros de Enfermagem, Registros Eletrônicos de Saúde

## Abstract

**Objective:**

To implement, on health management software, electronic records of the perioperative nursing process and the stages of transoperative and immediate postoperative nursing diagnoses, based on the NANDA International taxonomy.

**Method:**

Experience report conducted from the completion of the *Plan-Do-Study-Act* cycle, which allows improvement planning with a clearer purpose, directing each stage. This study was carried out in a hospital complex in southern Brazil, using the software Tasy/Philips Healthcare.

**Results:**

For the inclusion of nursing diagnoses, three cycles were completed, predictions of expected results were established, and tasks were assigned, defining “who, what, when, and where”. The structured model covered seven possibilities of aspects, 92 symptoms and signs to be evaluated, and 15 nursing diagnoses to be used in the transoperative and immediate postoperative periods.

**Conclusion:**

The study allowed implementing electronic records of the perioperative nursing process on health management software, including transoperative and immediate postoperative nursing diagnoses, as well as nursing care.

## RESUMO

**Objetivo:**

Implementar, em um *software* de gestão em saúde, os registros eletrônicos do processo de enfermagem perioperatório e a etapa de diagnósticos de enfermagem transoperatório e pós-operatório imediato, fundamentados na taxonomia *NANDA International*.

**Método:**

Relato de experiência conduzido a partir da realização do ciclo PDSA (*Plan-Do-Study-Act*), o qual permite o planejamento de melhoria com um propósito mais claro, direcionando cada etapa. Este estudo foi realizado em um complexo hospitalar da região sul do Brasil, utilizando o *software Tasy/Philips Healthcare*.

**Resultados:**

Para a inclusão dos diagnósticos de enfermagem, rodaram-se três ciclos, estabeleceram-se previsões de resultados esperados, e as tarefas foram atribuídas, definindo “quem, o quê, quando e onde”. O modelo estruturado contemplou sete possibilidades de aspectos, 92 sinais e sintomas a serem avaliados e 15 diagnósticos de enfermagem para serem utilizados no transoperatório e pós-operatório imediato.

**Conclusão:**

O estudo possibilitou implementar, em um *software* de gestão em saúde, os registros eletrônicos do processo de enfermagem perioperatório, compreendendo diagnósticos de enfermagem do transoperatório e pós-operatório imediato, além de cuidados de enfermagem.

## RESUMEN

**Objetivo:**

Implementar, en un *software* de manejo de la salud, registros electrónicos del proceso de enfermería perioperatorio y la etapa de diagnósticos de enfermería transoperatorios y postoperatorios inmediatos, con base en la taxonomía *NANDA internacional*.

**Método:**

Informe de experiencia realizado a partir de la consecución del ciclo *Plan-Do-Study-Act*), que permite planificar la mejora con un propósito más claro, dirigiendo cada etapa. Este estudio fue realizado en un complejo hospitalario en el sur de Brasil, utilizando el *Software Tasy/Philips Healthcare*.

**Resultados:**

Para la inclusión de diagnósticos de enfermería, se concluíran tres ciclos, se estableceran predicciones de los resultados esperados, y se asignaron tareas, definiendo “quién, qué, cuándo y dónde”. El modelo estructurado contempló siete posibles aspectos, 92 signos y síntomas para ser evaluados y 15 diagnósticos de enfermería para ser utilizados en el transperatorio y postoperatorio inmediato.

**Conclusión:**

El estudio permitió implementar, en un *software de* manejo de la salud, registros electrónicos del proceso de enfermería perioperatorio, que comprende diagnósticos de enfermería transoperatoria y postoperatoria inmediata, además de los cuidados de enfermería.

## INTRODUCTION

Perioperative nursing care is characterized by the care provided to the surgical patient during all phases of the immediate preoperative, transoperative, and intraoperative and immediate postoperative periods^(1)^. Concerning these phases, the 24 hours preceding the surgery are considered immediate preoperative; transoperative period starts when the patient is received in the operating room, going until the moment he/she is sent to the post-anesthesia care unit (PACU)^(1)^; the intraoperative period goes from the beginning to the end of anesthesia^(1)^; the immediate postoperative period is the first 24 hours after the anesthetic-surgical procedure. The Systematization of Perioperative Nursing Care (*SAEP*) is a methodology that encompasses nursing care in the perioperative period^(2)^.

In the United States of America (USA), the Association of periOperative Registered Nurses (AORN) recommends that operating room (OR) nurses use the standardized model called Perioperative Nursing Data Set (PNDS)^(3)^. In Brazil, the Federal Nursing Council (COFEN) Resolution no. 358/2009 recommends that all health care institutions providing nursing care implement and use the Nursing Care Systematization (*SAE*) methodology^(4)^. On SAEP, nurses are responsible for carrying out the Nursing Process (NP); therefore, they shall go through the five stages: Nursing History (NH), Nursing Planning, Nursing Diagnoses (ND), Implementation, and Evaluation. All stages are interrelated and take place concurrently^(4)^.

The ND stage is international standardization, based on NANDA-I^(5)^; it is an action private to the nurse and reflects the clinical judgment of the care needs identified. The nurse, supported by history taking and physical examination during the nursing records collection, provides the base for establishing interventions, which lead to the expected outcomes^(5)^. To ensure the continuity and quality of care, the information inherent to the nursing care process has to be recorded. Therefore, COFEN Resolution no. 429, of 2012, provides for the responsibility and duty of professionals to record them in the patient’s medical record, using traditional (paper) or electronic support^(6)^.

In health, the role of information and communication technologies in professional routine and their influence in the work processes is growing^(7)^. The use of electronic records is a reality in the Brazilian and global health scenarios^(8,9)^. Nurses perceive positive impacts on process agility and effectiveness, enhancing the improvement in resolution regarding the use or implementation of electronic systems, which is capable of accelerating the application of improvements and the creation of strategies in health facilities^(9)^. Aiming at mapping the ND of the NANDA-I taxonomy, through the analysis of medical records of patients in the transoperative period, a recently published study^(10)^ concluded that most diagnoses found during the intraoperative period were risk diagnoses.

The NP record, in Brazil, has to be carried out in all sectors where there is nursing care^(4)^. The NP documentation, mapped in a study carried out in São Paulo, showed that departments such as the OR are among those that do not record any documentation in the patient’s medical record^(11)^. Due to this difficulty, the research problem was defined: is there lack of electronic registration in health management software for the nurses to document the nursing process in the transoperative and immediate postoperative periods?

The definition of the transoperative and immediate postoperative periods is justified due to the OR nurses role in patient care. Thus, the objective of this study was to implement the stage of nursing diagnoses of the transoperative and immediate postoperative periods, based on the NANDA International taxonomy, in the perioperative nursing process electronic records of health management software.

The implementation of electronic records in the software is of paramount importance, as it facilitates the nursing process and its registration in the OR department, helping in care and providing better care planning for the surgical patient. Moreover, the importance of the nursing record duly documented with the use of the taxonomies is highlighted, as it facilitates communication among the team and other nursing professionals from other departments, or even among health professionals in general. Finally, it should be noted that nursing records ensure one of the patient’s rights, that is, access to information on their health data.

## METHODS

### Design of Study

This is an experience report, conducted after the completion of the PDSA (*Plan-Do-Study-Act*) cycle^(12)^. The PDSA helps the researcher focus on building knowledge, critical in the learning required to qualify an improvement^(13)^. In addition, the PDSA allows teams to plan for improvement, with a clearer purpose, and to direct each stage on the way^(12,13)^.

### Population

For this study, it was defined that the implementation of the perioperative electronic nursing record would initially be carried out for patients with size III and IV procedures. Size III is any procedure including surgeries that last more than four hours, up to the limit of six hours; size IV are surgeries lasting more than six hours^(14)^. The classification refers to nursing care hours and the operating room usage time^(14)^. That definition is explained by the particularities and complexities of each Operating Room of the facility where the research was conducted, the profile of patients treated, and the team size in the OR.

### Local

The study setting was the oldest hospital in the state of Rio Grande do Sul/Brazil, and one of the most modern hospital complexes in the country^(15)^, contemplating the surgical segment. The hospital complex consists of seven hospitals, 10 OR to serve the areas of general surgery, cardiology, neurosurgery, pneumology, oncology, pediatrics, and transplants, with a total of 57 operating rooms. The institution’s performance indicators, in 2020, point to approximately 45 thousand surgical procedures. The production was lower than in previous years, due to the outbreak of the pandemic caused by the SARS-CoV-2 virus in 2020, which led to restrictions recommended by the World Health Organization (WHO) and the Brazilian Health Regulatory Agency (ANVISA)^(16,17)^.

### Selection Criteria

Inclusion criteria for the selection of nurses were: being a nursing supervisor, being a clinical nurse with at least two years of experience in the surgical area, and/or having a graduate certificate. Such criteria aimed at knowing surgical nursing care in theory and practice. Furthermore, a systems analyst nurse, with experience of more than two years in the management of software was included in the group due the system specificity. The only exclusion criterion was the individual not participating in all stages of the process.

The sample was intentionally constituted, comprising a nursing supervisor of an operating room, two clinical nurses, a systems analyst nurse, two professors from the Nursing Master’s program at UFCSPA, and an undergraduate research project scholarship holder, totaling seven participants.

### Electronic Health Records Management Software

The product of this study was implemented in health management software, called *Tasy/Philips Healthcare* ^(18)^, used for electronic records. The software is divided into modules, such as: invoicing, financial, managerial, assistance, among others. It is currently developed in Java, which provides a user-friendly interface. The NP was implemented in the *Tasy* in 2017^(19)^ and is used in the institution where the study was carried out. The system brings, in its structure, the profile “ENF – Nurse” (profile used by nurses in the inpatient units, ICUs and emergency), “ENF – Nurse at the Operating room w/SP” (profile used by nurses in the surgical service, who provide care with a satellite pharmacy), and “ ENF – Operating Room Surgical Center Nurse w/o SP” (profile used by the surgical service nurse, who provides care without a satellite pharmacy). In these profiles, nurses can register their nursing records in the “Patient’s Electronic Record – *PEP*” or in the “Perioperative Electronic Record – *PEPO*”. In 2017, *SAE* was structured in the “Patient’s Electronic Record – *PEP*” profile. In the profile “ENF – Operating Room Nurse w/SP or w/o SP”, there is the tab Surgery Management.

The results generated by this study were implemented in the system used by the health institution and are available to nurses who work in the operating room area.

### Data Collection

This stage of the study was carried out from May to October 2020, after the project being approved by the Research Ethics Committee. The descriptive aspects of each of the PDSA cycles, pertinent to the execution of this study, are presented below.

PLAN: this stage is the moment when the study participants meet the researchers to “plan” the change. In this stage, the objective is defined, the plan for executing the cycle is designed (who, what, where, when) and the plan for data collection is carried out^(12–20)^. A cycle execution plan was then prepared: the system was verified, aiming at understanding and evaluating the possibilities of the *Tasy*, and the particularities of the material elaboration were defined and later inserted in the system.

*DO*: contemplates plan execution, the *“*to do”. It is also the time to document problems, unexpected observations, and to include aspects that were not part of the plan^(12,20)^. At this stage, for the construction and elaboration of the material, NDs listed from an integrative review, carried out by the researchers at the beginning of the research, considering this the first cycle, were presented^(21)^.

*STUDY*: allows studying, complementing data analysis and considerations made by the participants and researchers^(12,13)^. At the “*study*” cycle, a detailed review of the model was carried out to check whether any data was outside the proposed standards, meeting the needs of the proposed improvement.

ACT: consists of the action based on what was learned in the previous stages; and, if necessary, a new cycle is planned to test and implement changes^(12,13)^. In view of this, the use of NP in electronic records in practice began, as well as a new cycle for refining interventions.

### Data Analysis and Treatment

The team of nurses from all the operating rooms of the hospital started to use, in practice, the process implemented in the system *Tasy*. To assist in the dissemination of knowledge and the use of this process in the system, a tutorial video was developed, contemplating the accomplishment of the task step by step. The perioperative NP electronic records were designed based on a nursing assessment model, divided into the two areas in which the OR nurse works: the transoperative period, comprising the entire intraoperative period, and the immediate postoperative period, following aspects to be evaluated, defining characteristics, and suggested ND.

### Ethical Aspects

This study followed ethical and legal principles at all stages, as provided for in Resolution 466/12 of the National Health Council^(22)^. It was approved by the University Research Ethics Committee, submitted with opinion number 4.457.425 in the year 2020, and the hospital institution was a co-participant in the research.

## RESULTS

**Chart 1.**
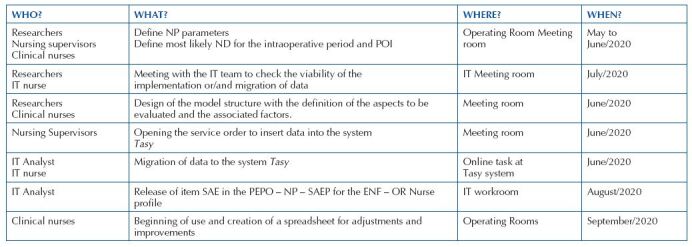
Plan for implementing the PDSA cycle: task distribution. Source: Prepared by the authors – Porto Alegre, RS, Brazil, 2021.

The results are presented according to the steps of the PDSA cycles described below:

In the first stage, “Plan”, meetings were held with participants and researchers. After the meetings, the information was organized, the tasks distributed and described, as shown in Chart 1, following the proposed execution plan.

**Figure 1.**
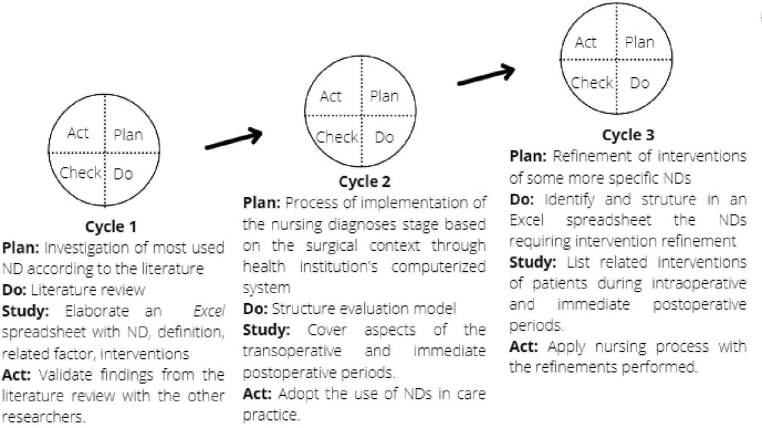
Use of cycles to implement the perioperative nursing process in the system *Tasy*. Source: Prepared by the authors – Porto Alegre, RS, Brazil, 2021.

The PDSA cycles applied to manage the change related to the development of this work product are presented below. An integrative literature review was considered^(21)^ as the initial learning cycle for implementing improvements in the perioperative nursing process. The three cycles are briefly described in Figure 1.

**Figure 2.**
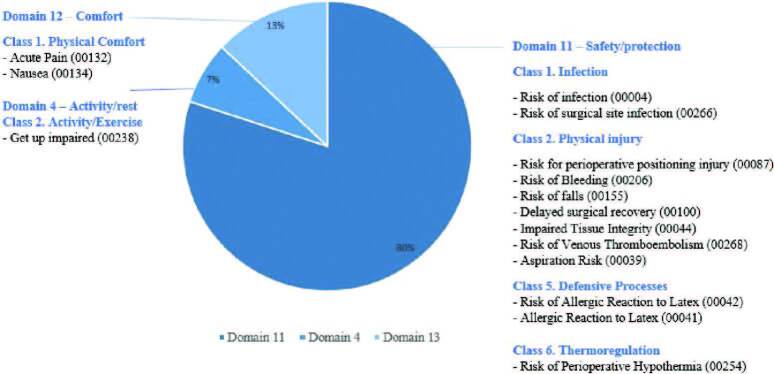
Nursing diagnoses, according to NANDA-I^(5)^, validated by the group of surgical nurses for this research.Source: Prepared by the authors – Porto Alegre, RS, Brazil, 2021.

In the second cycle, the NDs listed from the integrative review^(21)^ (marked with an asterisk) were presented to the research group; in addition, seven more NDs were suggested by the team of research nurses. It was completed with 15 NDs validated by the research team (Figure 2), later entered into the system.

**Figure 3.**
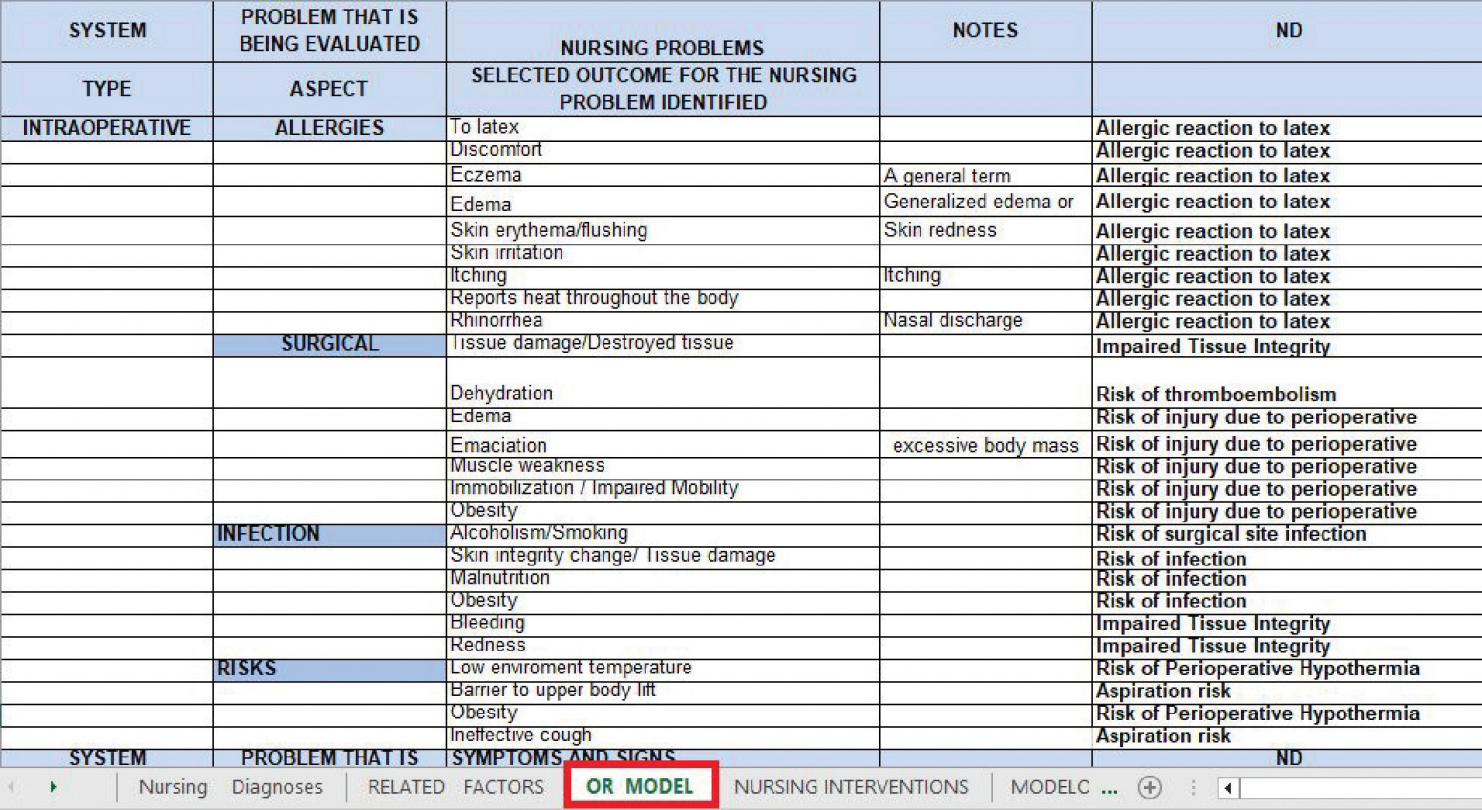
Structure of the nursing assessment model related to the transoperative period, organized in an *Excel* spreadsheet to be entered into the system *Tasy*. Source: Prepared by the authors – Porto Alegre, RS, Brazil, 2021.

With the information obtained in the meeting with the Information Technology (IT) team, regarding the possibilities that the system *Tasy* provided, and following the reasoning used in the implementation of electronic records related to ND in the remaning sections of the institution, a nursing assessment model was created to specifically assist patients in the periods of their surgical experience. Based on this model, organized in an Excel spreadsheet, the system indicates the NDs to the nurses. For its elaboration, the columns were filled as follows: type (intraoperative), aspect (allergies, surgical positioning, infection, risks, etc.) and the ND to be suggested according to the defining characteristics (Figure 3). The structured model included seven possible aspects, 92 symptoms and signs to be evaluated, and 15 suggestions for associated NDs.

Based on the opening of the service order (*OS*), the IT assistant, assigned to the task, performed operations and activities related to data entering from tables into the system *Tasy*. Once the transition of information in the Oracle database of software *Tasy* was completed, it was observed that each ND was linked to the respective related factors previously registered in the system.

Right at the beginning of the process application, the team of clinical nurses identified the need to insert nursing care related to four more specific NDs of the intraoperative period, namely: Risk of Allergic Reaction to Latex; risk from perioperative positioning injury; Thromboembolism risk; and Risk of perioperative hypothermia.

**Figure 4.**
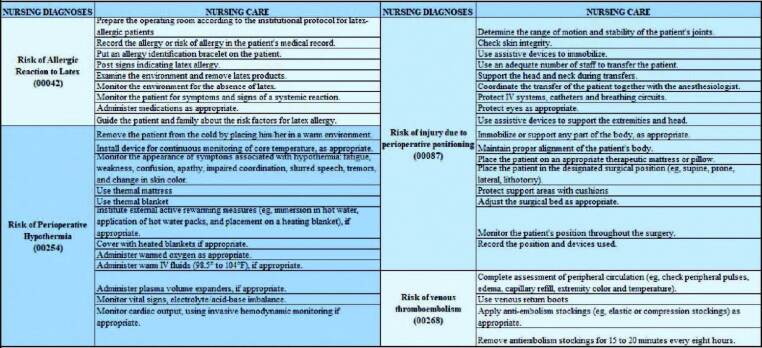
Nursing care related to the transoperative period, organized in an Excel spreadsheet to be entered into the system *Tasy*. Source: Prepared by the authors – Porto Alegre, RS, Brazil, 2021.

The third cycle was performed: the group of researchers met and 41 new interventions, according to the NIC (Figure 4), were listed in a table, aiming at refining the care plan for the profile of patients. Subsequently, the table was sent to the IT systems analyst and entered into the system *Tasy*.

The institution’s OR nursing team began to use, in practice, the process implemented in the software. The system allows nurses to carry out NP stages by clicking on the “*SAE*” item in the patient’s perioperative electronic record. To start registration, click on “new”, selecting the “Nursing Process – SAEP”, in the field “model” and the evaluation matrix based on symptoms and signs opens, which suggests ND according to the selection. The institution’s working group decided to keep the item with the name “Nursing Process – SAEP”, considering that this is the nomenclature previously proposed and recognized by the Brazilian Association of Operating Room Nurses, Anesthetic Recovery and Material and Sterilization Center (SOBECC)^(1)^.

The nurse has the possibility to select symptoms and signs according to the assessment performed on the patient. For these symptoms and signs, the system suggests the NDs. If a wrong selection of a sign or symptom is performed, the system allows the nurse to “exclude the selected result”.

Subsequently, the nurse shall click on the lower options bar “Diagnoses” and is sent to a new screen, where he/she can view the NDs from the previously analyzed aspects in yellow. In this stage, it is possible to confirm the NDs of choice, select the related factor and, then, click with the left mouse button on the diagnosis and select the option “confirm diagnosis”. If necessary, the system allows the nurse to exclude a ND, selecting it and clicking on the “Disconfirm diagnosis” option.

After confirming the ND appropriate for the patient, the nurse must select the option “generate nursing interventions”. The system itself suggests nursing care. The nurse shall then read the options provided by the system, evaluating which interventions will be appropriate for the moment of patient care, with the possibility of excluding those that are not in accordance with the care practice in the intraoperative or immediate postoperative period at the PACU.

## DISCUSSION

To achieve the objective outlined in this research, it was necessary to adapt the existing nursing assessment model in the system *Tasy* ^(19)^, continuing another survey previously carried out^(19)^. Therefore, the model mentioned was modified, in a succinct and specific way, focusing on transoperative and immediate postoperative care, listing the aspects that contemplate the patient’s needs, facilitating the work of the nurse, suggesting the ND and interventions relevant to the patients’ care according to their needs.

These improvement actions, identified as necessary in a recent study carried out at the institution, contribute to the nurses’ work and reflect improvements in care practices^(23)^. The use of electronic health records in specialized perioperative nursing care may improve nurses’ documentation practice^(24)^.

Currently, in the institution’s *Tasy* system, where the product was implemented, the nurse has access to all NANDA-I NDs, either through the use of SAE model in the “Patient’s Electronic Record – PEP”, or specifically for patients in the surgical process, with the realization of the SAEP model in the “Perioperative Electronic Record – PEPO”. The viability of carrying out the SAEP meets the good practices advocated by SOBECC^(1)^. The use of specific models, such as the “Perioperative Patient-Focused Model”, suggested by AORN, guides the conceptual framework for perioperative nursing practice, highlighting as its strength the application of a results-oriented process^(25)^. A study carried out in the state of Piauí shows the importance of the nurse’s presence in the intraoperative process, providing supervised care, ensuring safety to the surgical patient^(24)^.

The implementation of SAEP is a challenge for nurses, although it is a tool to make care individualized and effective^(26)^. It allows the interaction of nurses in the perioperative process, planning individualized care, and focused on a process with a scientific nature^(26)^, aiming at quality. Danish researchers point out that education, changes in habits, and culture are essential to increase perioperative nurses documentation practice and to improve patient’s safety^(27)^.

One issue that interferes with the full applicability of the NP is personnel dimensioning. With headcount limitation, the team ends up prioritizing care tasks, and nursing records remain in the background. The Federal Nursing Council (COFEN) establishes the number of one nurse for every three elective surgery rooms within 24 hours, and an exclusive nurse in the urgency/emergency room, depending on the size of the surgery and the level of complexity^(14)^.

The number of nursing professionals in the OR, mainly nurses, has to be adequate as suggested for the sizing, as nurses provide various administrative tasks and do not directly follow the patient during the anesthetic-surgical procedure^(23)^. Due to this deficiency, this study group determined that, intraoperatively, the computerized NP shall be performed for all patients in surgeries of size III and/or IV initially, not contemplating procedures of size I and II. Regarding PACU, during the immediate postoperative period, the NP shall be performed for overnight patients and for those with a request for hospitalization who remain waiting for a bed – at this first moment, not contemplating outpatients with a quick visit to PACU. In some ORs, where the demand is lower, it is possible to carry out NP for all patients; however, as the realities are different, in other ORs, where the demand is greater, partial NP takes place, that is, nursing is divided between care tasks and administrative tasks, having to prioritize the performance of direct patient care. Nursing management and coordination shall assess the scenario, seeking balance between the ideal and the real.

To monitor progression, the performance of the applicability, and the quality of this product in the assistance quantitatively, a service order was opened, together with the IT team, requesting the creation of an SAEP indicator, on the platform *Power BI.* The use of indicators represents a support for nursing practice, a continuous monitoring of the quality of the service provided, as well as a tool to support management^(28)^.

The limitations found during the study are related to the lack of knowledge on the part of some nurses with older training, who did not seek updates on care practices or computerized issues related to registration of electronic medical records. Thus, some professionals’ lack of practice and skill on the use of technology hindered the adherence to the use of the electronic health system. The developed video tutorial, contemplating the step-by-step stages to carry out the task, helped in the dissemination of knowledge for the use of this process (the video is published on YouTube and is still accessed). Currently, this video is used by the institution to train new employees, before they start their activities in the operating room.

For nurses at the health institution that was the object of the study, the reality of the nursing record had its practical change evidenced, with the implementation and use of the software. Before the study, there was no nursing record in place; after the study and its implementation, the nurses began to record the NP electronically.

The model created had a theoretical basis, as well as the practical participation of specialists. Based on that, nurses can select nursing diagnoses according to the needs of each individual patient, providing appropriate interventions, and using standard language, thus allowing assertive communication.

Another relevant aspect to be considered is that communication between nurses was facilitated by the existence of electronic records, and are more effective in transferring information about patients, throughout their journey, to other departments of the institution, such as the inpatient unit.

Finally, it should be noted that the implementation of records requires computer skills and dedication of professionals’ time. Those involved in this study, as well as the institution, worked with training for professionals, so that they could improve the use of software and also clarify possible doubts.

## CONCLUSIONS

The study allowed implementing electronic records of the perioperative nursing process on health management software, including transoperative and immediate postoperative nursing diagnoses, as well as nursing care. Computerization can contribute to the improvement of this process, since a structured model was implemented, which included aspects, nursing problems and nursing diagnoses already described in the system, contributing to the improvement of care practices.

Nurses need to have the ability, technical capacity, and scientific knowledge to track the real and potential nursing diagnoses, based on the individual’s needs in a systematic and individualized way. The continuation of studies related to the topic is recommended, with working groups that promote discussions about necessary updates, related to the amount of data, and maintenance of the system update according to new NANDA-I standards.
